# A Method for Consensus Reaching in Product Kansei Evaluation Using Advanced Particle Swarm Optimization

**DOI:** 10.1155/2017/9740278

**Published:** 2017-02-12

**Authors:** Yan-pu Yang

**Affiliations:** School of Construction Machinery, Chang'an University, Xi'an 710064, China

## Abstract

Consumers' opinions toward product design alternatives are often subjective and perceptual, which reflect their perception about a product and can be described using Kansei adjectives. Therefore, Kansei evaluation is often employed to determine consumers' preference. However, how to identify and improve the reliability of consumers' Kansei evaluation opinions toward design alternatives has an important role in adding additional insurance and reducing uncertainty to successful product design. To solve this problem, this study employs a consensus model to measure consistence among consumers' opinions, and an advanced particle swarm optimization (PSO) algorithm combined with Linearly Decreasing Inertia Weight (LDW) method is proposed for consensus reaching by minimizing adjustment of consumers' opinions. Furthermore, the process of the proposed method is presented and the details are illustrated using an example of electronic scooter design evaluation. The case study reveals that the proposed method is promising for reaching a consensus through searching optimal solutions by PSO and improving the reliability of consumers' evaluation opinions toward design alternatives according to Kansei indexes.

## 1. Introduction

As the international market is globalizing at high speed, new opportunities have been opened up for businesses with successful new products. In an era of highly competitive and uncertain market, companies that listen to their consumers are more likely to be successful [1]. With the increasing trend of emotional consumption, consumers making purchase decisions is no longer merely a consideration regarding function or practical use, but a comprehensively and perceptually assessment process through sense perception [2]. Numerous studies have been conducted to gain depth and insight into consumers' subjective perception about products, among which Kansei engineering (KE) is the most notable [3–5]. Covering the meanings of sensibility, impression, and emotion, Kansei means all the senses of an individual's subjective impression and recognition from a certain artifact, environment, or situation, as described by Nagamachi [5]. It is associated with consumers' physiological and psychological feelings, and if properly implemented in product development, promotion in consumers' satisfaction will be great. For decades, KE has been developed as a consumer oriented technique and connected to the industrial world to create numerous successful products and innovations [6]. This method involves five types [3, 7]: (1) category classification for identifying consumer's affective need with a tree structure; (2) Kansei engineering system (KES), a computer aided system with mathematically statistical tools to connect Kansei and product properties; (3) hybrid engineering system, incorporating KES and Kansei prediction elicited by product properties; (4) KE modeling for assessing consumers' feeling of Kansei words; (5) virtual KE with a virtual reality system for presenting products and standard data collection systems. Therefore, KE is often associated with emotional engineering that can link consumers' emotions to product properties so as to determine and evaluate new design solutions. KE has been proven to be effective and successfully found its application in a wide range of industry, such as chairs [8], car steering wheels [2], alarm clocks [9], disposable razors [10], and sunglasses [11]. KE also permeates into other design fields and yields many brilliant results, for example, packing design [12], logistics services [13], trade show booth design [14], user interface design [15, 16], and so on.

When planning a product development process, concept evaluation is often involved as it facilitates the assessment of the overall feasibility of design alternatives. Through concept evaluation, both time and cost can be saved as 70%–80% of the final product quality and 70% of the product entire life-cycle cost are committed in the early product design phase [17]. Design concepts resulting from poor selection without comprehensive concept evaluation may lead to large redesign costs because it can hardly be compensated in the later stage [18]. When it comes to product appearance design, the importance of Kansei evaluation is obvious as almost every company has laid increasing concern on satisfying customer needs [19], and primary importance should be attached to both functional and affective needs [2]. In this regard, evaluating consumers' perception and recognition of a product design alternative will add additional insurance and reduce uncertainty to successful product design.

Product Kansei evaluation is a systemic and important step in determining consumers' preference using Kansei criteria against product design alternatives. The main objective is help to determine that the final design solution reflects consumers' subjective preference and can reach a certain degree of satisfaction and consensus. To achieve this goal, three procedures should be conducted: (1) obtaining Kansei data for the products to be evaluated; (2) making assessment by consumers who are the users or potential users with semantic differential (SD) method associated with statistical analysis; (3) consensus computing and reaching process. Collecting Kansei words and extracting primary Kansei attributes are the basic principle for getting valid results in Kansei evaluation [20], methods involving linear inference techniques (mainly referring to statistical methods, e.g., correlation coefficient analysis, principal component analysis, factor analysis, and multiple regression analysis) and nonlinear inference techniques (e.g., genetic algorithms, neural networks, and fuzzy logic). Nevertheless, due to different background and social experience, there is inconsistency existing in consumers' cognition and their preference may vary from person to person, which may stunt consensus reaching. Here consensus refers to agreement on a product design option by most members of a Kansei evaluation team despite their different opinions [21]. If valuators' preferences and opinions were simply aggregated without considering their consensus in evaluation process, it may result in a lower acceptability of the obtained solution [22]. The better the solution is, the greater the agreement this solution generates among valuators [23]. As unanimous agreement is hard to reach, “soft” consensus measures which reflect all possible partial agreement are often adopted to gauge the cohesiveness among valuators' opinions [24]. In consensus process, one of the most significant issues is how to guide decision makers to reach consensus with minimum adjustments, toward which two rules should be obeyed [25]: minimizing the distance between the original opinions and adjusted opinions; minimizing the number of adjusted preference values. Essentially, this is an optimization problem and various optimization methods can be used for seeking optimal solution, like neural network (NN), support vector machine (SVM), genetic algorithms, and particle swarm optimization (PSO). However, very few studies focus on employing optimization methods for consensus reaching, and most researchers lay emphasis on studying consensus model for effective communication and feedback. While in product Kansei evaluation process, consumers' sensation and perception against a product is conveyed more through first impression than rational analysis. Hence communication may be an ineffective method and will affect efficiency if there are a large number of people involved in Kansei evaluation. Based on this point, given the advantage of global optimization with simple operation and parallel search comparing with other optimization approaches [26], we employed PSO to seek for consensus reaching with minimum adjustments in product Kansei evaluation.

The rest of this paper is organized as follows: Section 2 displays the methods for consensus reaching in product Kansei evaluation, including consensus measurement model, advanced PSO for adjusting consumers' opinions, and analysis of product Kansei evaluation procedures. Then, a numerical example is provided to illustrate the detailed implementation of the proposed method in Section 3. Finally, we summarize and highlight the contribution of this paper.

## 2. Methods

### 2.1. Consensus Measurement Model

Let *E* = {*e*_1_, *e*_2_,…, *e*_*m*_} (*m* ≥ 2) be a set of consumers, whose weighting vector is *λ* = {*λ*_1_, *λ*_2_,…, *λ*_*m*_}^*T*^ with *λ*_*i*_ ≥ 0, *i* = 1,2,…, *m*, and ∑*λ*_*i*_ = 1. The consumers are invited to participate in product Kansei evaluation about a set of product design alternatives *X* = {*x*_1_, *x*_2_,…, *x*_*t*_} (*t* ≥ 2). Assume that the set of Kansei evaluation indicators is *C* = {*c*_1_, *c*_2_,…, *c*_*n*_} (*n* ≥ 2) and the weighting vector is *W* = {*w*_1_, *w*_2_,…, *w*_*n*_}^*T*^ with *w*_*k*_ ≥ 0, *k* = 1,2,…, *n*, and ∑*w*_*k*_ = 1. In evaluating and decision making process, consensus threshold is often used for “soft” consensus measurement. Set the consensus threshold for *δ*. If the level of approval is above *δ*, then the evaluation result is highly reliable. Otherwise, measures should be taken to adjust consumers' opinions for consensus improving.

Let vector *A*_*ij*_ = (*a*_1*ij*_, *a*_2*ij*_,…, *a*_*nij*_) represent the set of scoring of *x*_*j*_ by *e*_*i*_ according to *C*, and then we can get an evaluation matrix of *x*_*j*_, *A*_*j*_ = [*A*_1*j*_, *A*_2*j*_,…, *A*_*mj*_]. Normalization is computed as follows:

(1) $$\begin{array}{c} a_{kij}^{\prime }=\frac{a_{kij}-\min_{1\leq k\leq n}\min_{1\leq i\leq m}a_{kij}}{\max_{1\leq k\leq n}\max_{1\leq i\leq m}a_{kij}-\min_{1\leq k\leq n}\min_{1\leq i\leq m}a_{kij}}. \end{array}$$

Euclidean distance is used to calculate the opinion difference between consumer *h* and consumer *l*:

(2) $$\begin{array}{c} D\left(\;A_{hj}^{\prime },A_{lj}^{\prime }\;\right)=\sqrt{\overset{n}{\underset{k=1}{\sum }}w_{k}{\left(a_{khj}^{\prime }-a_{klj}^{\prime }\right)}^{2}}. \end{array}$$

Then, the similarity of opinions by consumer *h* and consumer *l* is

(3) $$\begin{array}{c} S\left(\;A_{hj}^{\prime },A_{lj}^{\prime }\;\right)=1-D\left(\;A_{hj}^{\prime },A_{lj}^{\prime }\;\right). \end{array}$$

Consensus measurement of consumers' opinions upon *x*_*j*_ can be depicted by

(4) $$\begin{array}{c} {CON}_{j}=\frac{\sum_{h=1}^{m}\sum_{l=1}^{m}\mu \left(h,l\right)}{\sum_{h=1}^{m}\sum_{l=1}^{m}\lambda_{hl}}, \end{array}$$

where *μ*(*h*, *l*) = {*λ*_*hl*_, *S*(*A*_*hj*_′, *A*_*lj*_′) ≥ *δ*; 0, *S*(*A*_*hj*_′, *A*_*lj*_′) < *δ*}, and *λ*_*hl*_ is used for evaluating the joint weights of *h* and *l* with *λ*_*hl*_ = (*λ*_*h*_ + *λ*_*l*_)/2. Specially, if *h* = *l*, then *λ*_*hl*_ = *λ*_*h*_ = *λ*_*l*_ = *μ*(*l*, *l*).

Finally, the total score of *x*_*j*_ by consumers' evaluation can be computed by

(5) $$\begin{array}{c} P_{j}=\lambda A_{j}^{\prime }W^{T}. \end{array}$$

### 2.2. Advanced PSO for Consensus Reaching by Adjusting Consumers' Opinions

It is likely not to reach a consensus easily due to cognitive difference among consumers. Therefore appropriate measures should be taken to seek consensus with minimum adjustment of consumers' opinions, among which particle swarm optimization (PSO) is chosen here as it makes few or no assumptions about the problem being optimized and can search very large spaces of candidate solutions by iteration [26].

By asking consumers who participate in the evaluation process, the upper limit and lower limit of each consumer's opinion can be gained, between which consumers' opinions will be adjusted for consensus reaching. The upper limit vector and lower limit vector are shown as follows:

(6) Aju=A1juT,A2juT,…,AmjuTTAjl=A1jlT,A2jlT,…,AmjlTT,

where *A*_1*ju*_^*T*^, *A*_2*ju*_^*T*^,…, *A*_*mju*_^*T*^ and *A*_1*jl*_^*T*^, *A*_2*jl*_^*T*^,…, *A*_*mjl*_^*T*^ represent the upper limit and lower limit of adjusted opinions of *x*_*j*_ by consumers *e*_1_, *e*_2_,…, *e*_*m*_, respectively.

To seek the optimal solution in PSO, each candidate solution, called a particle, flies in the *N*-dimensional search space according to a speed. Suppose that there are *M* particles in the swarm, and then particle *p*_*j*_ has a position *p*_*j*_ = (*p*_1*j*_^*T*^, *p*_2*j*_^*T*^,…,*p*_*mj*_^*T*^)^*T*^ and a velocity *v* = (*v*_1*j*_, *v*_2*j*_,…, *v*_*mj*_), where *p*_1*j*_^*T*^, *p*_2*j*_^*T*^,…, *p*_*mj*_^*T*^ represent the automatically adjusted opinions of consumers *e*_1_, *e*_2_,…, *e*_*m*_. The velocity decides the flying distance and direction, and (4) is used as target optimization function. Thus the velocity and location updating of a particle can be calculated as follows:

(7) vαβt+1=ωvαβt+c1r1βtpbestαβt−xαβt+c2r2βtgbestβ−xαβtxαβt+1=xαβt+vαβt+1,

where *t* is the iteration number; *v*_*αβ*_(*t*) and *x*_*αβ*_(*t*) represent the velocity and position of particle *α* in the *β* dimension, respectively; *pbest*_*αβ*_(*t*) is the current best position of particle *α*; *gbest*_*β*_ shows the best fit that any particle of the swarm has ever achieved; *r*_1*β*_(*t*) and *r*_2*β*_(*t*) are two random numbers ranging from 0 and 1; *c*_1_ and *c*_2_ are two positive constants, denoting the cognitive and social components, respectively; *ω* is the inertia of the particle which is employed to improve the convergence of the swarm. Linearly Decreasing Inertia Weight (LDW) is often used to enhance the global exploration ability for searching in a larger space by increasing the value of *ω* when the evolution speed of the swarm is fast and maintain the particles searching in a small space to find the optimal solution more quickly by decreasing the value of *ω* if the evolution speed of particles slows down. *ω* can be calculated as follows [27]:

(8) $$\begin{array}{c} \omega =\omega_{max}-\frac{\omega_{max}-\omega_{min}}{t_{max}}\times t, \end{array}$$

where *ω*_max_ and *ω*_min_ represent the maximum and minimum of *ω*, respectively. Generally, *ω* linearly decreases from 0.9 to 0.4.

*gbest*_*β*_ is the mapping of adjusted evaluation matrix *A*_*j*_ and can be calculated as

(9) $$\begin{array}{c} gbest_{\beta }=a_{\left\lceil \beta /n\right\rceil j}, \end{array}$$

where ⌈⌉ denotes a ceiling function to map a real number to the smallest following integer.

**Figure 1:**
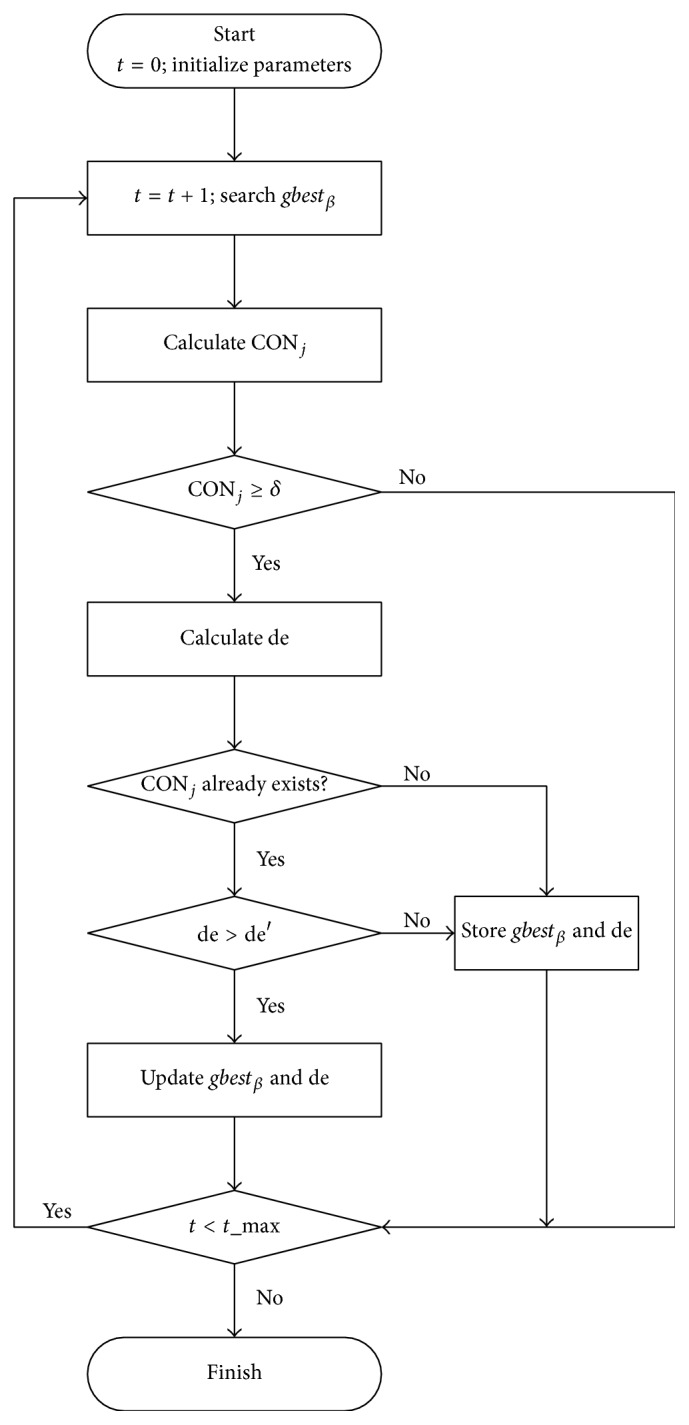
The procedure for consensus reaching with PSO.

Set *t*_max as the maximal iteration generations of PSO. When *t* = 0, initialize all particles with constraint of *A*_*ju*_ and *A*_*jl*_. Then the procedure for consensus reaching by automatically adjusting consumers' opinions with PSO can be divided into five steps, depicted in Figure 1.

**Step 1 .**

If *t* < *t*_max, then replace *t* with (*t* + 1) and search the global best position *gbest*_*β*_ in generation *t* using (8); otherwise finish the iteration.

**Step 2 .**

Calculate consensus of *gbest*_*β*_ using (4). If CON_*j*_ ≥ *δ*, then store *gbest*_*β*_ and go to Step 3; otherwise go back to Step 1.

**Step 3 .**

Calculate the deviation de between *gbest*_*β*_ and *A*_*j*_ using (2) and (3). Compare consensus of *gbest*_*β*_ in Step 2 with that from generation 1 to generation (*t* − 1) and decide whether it is equal to the previous consensus or not. If the condition is met, mark and store the adjusted evaluation matrix with *gbest*_*β*_′, and go to Step 4; otherwise store it with *gbest*_*β*_ and the corresponding de, and go back to Step 1.

**Step 4 .**

Calculate the deviation between *gbest*_*β*_′ and *A*_*j*_, representing with de′. If de > de′, then go to Step 5; otherwise go back to Step 1.

**Step 5 .**

Update *gbest*_*β*_ with *gbest*_*β*_′ and de with de′. Go back to Step 1.

The minimal deviation between the set of *gbest*_*β*_ and primary *A*_*j*_ will be selected as the optimal solution and adjusted opinions of consumers. Then the total score of product design alternative *x*_*j*_ can be computed with (5).

## 3. Case Study

**Figure 2:**
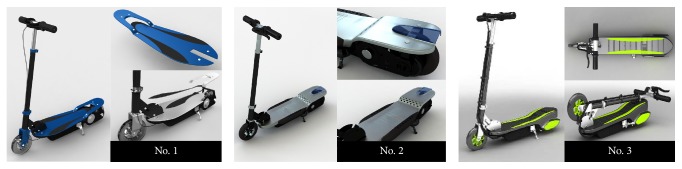
Design solutions.

A case study of electric scooter was used to determine the proposed method's ability for reaching consensus in product Kansei evaluation process. The author's previous study has gained six primary Kansei needs through investigating and clustering about the target product, seen in [1]. Each Kansei adjective was given the antonym and a seven-point Likert scale was used to evaluate customers' response about product design alternatives, shown as follows: (1) untechnical–technological; (2) inactive–dynamic; (3) outdated–futuristic; (4) feminine–manly; (5) dimmed–vivid; (6) partial–integral. Three industrial designers were asked one each to create a design solution according to consumers' Kansei needs (seen in Figure 2) and 9 consumers (4 middle school students and 5 high school students) were randomly selected and invited to give their score about the alternative solutions according to the Kansei indexes (seen in Tables 1, 2, and 3).

Consumers who participated in the evaluation process weight equally and weights of the six Kansei indexes can be calculated using AHP method [28], and the results are as follows: technological (0.25), dynamic (0.22), futuristic (0.18), manly (0.09), vivid (0.12), and integral (0.14). Consensus threshold value was set as *δ* = 0.7, and then Kansei evaluation consensus of each design option can be calculated using (1)–(4). Programmed in MATLAB software, consensus of No. 1, No. 2, and No. 3 was calculated with the results of 0.531, 0.630, and 0.704, respectively. It can be seen that the consensus results of No. 1 and No. 2 cannot meet the requirements and PSO should be employed to adjust consumers' opinions for consensus reaching.

Generally, particle swarm size ranges from 10 to 50 depending on different applications and problems [29]; and here it is equal to 20. *c*_1_ and *c*_2_ belong to the range of [0, 4], and *c*_1_ = *c*_2_ = 2 may be preferable [27]. *t*_max is set to 500. By asking consumers' advice, the adjustment space of consumers' opinions is set to [−1, 1]. Yet the adjusted evaluation value should fall in the range of −3 and 3. Five feasible solutions of No. 1 were found (seen in Table 4) and the optimal solution was gained with the minimum deviation (0.064), and the result is shown in Table 5. Similarly, feasible and optimal solutions of No. 2 were calculated, seen in Tables 6 and 7.

Using (5) to compute the scores of each design solution and comparing the result of considering evaluation consensus without considering consensus, detailed results were obtained, shown in Table 8. We can see that, through consensus reaching process and adjusting consumers' opinions, consensus value and score of each design alternative are improved without affecting the overall evaluating results, which would improve the reliability of consumers' opinions. Thus the three design alternatives can be ranked in descending order as No. 3 ≻ No. 1 ≻ No. 2.

## 4. Conclusion

A novel method for consensus reaching in product Kansei evaluation process using advanced particle swarm optimization (PSO) algorithm is proposed in this work. The method demonstrates the capacity and efficiency for reaching consensus by minimizing the adjusted opinions of consumers. An advanced PSO algorithm is presented combined with Linearly Decreasing Inertia Weight (LDW) method to enhance the global exploration ability for searching in a larger space when the evolution speed of the swarm is fast and maintain the particles searching in a small space to find the optimal solution more quickly if the evolution speed of particles slows down. The process of the proposed method is discussed and illustrated using an example of electronic scooter design evaluation for consensus reaching. The results suggest that using advanced PSO helps to reach a consensus and find the optimal solutions with minimal adjustment of original evaluation value and improve the reliability of consumers' evaluation opinions toward design alternatives according to Kansei indexes. It appears that the proposed method is promising for reaching a consensus in product Kansei evaluation process.

## Figures and Tables

**Table 1:** Scores of No. 1.

Consumers	Kansei indexes					
	Untechnical–technological	Inactive–dynamic	Outdated–futuristic	Feminine–manly	Dimmed–vivid	Partial–integral
1	3	2	2	3	3	−2
2	2	1	3	2	2	1
3	2	3	0	−1	2	1
4	1	2	2	2	2	2
5	−2	3	3	3	2	−2
6	2	2	2	3	3	1
7	3	2	−2	2	2	2
8	2	2	2	0	1	1
9	2	2	1	3	2	2

**Table 2:** Scores of No. 2.

Consumers	Kansei indexes					
	Untechnical–technological	Inactive–dynamic	Outdated–futuristic	Feminine–manly	Dimmed–vivid	Partial–integral
1	0	2	1	1	−2	3
2	1	1	1	2	−1	2
3	−1	2	0	1	0	0
4	1	1	1	1	−1	1
5	2	1	2	2	−1	1
6	−2	2	1	2	−2	2
7	0	1	−1	2	1	2
8	1	2	−2	3	1	−1
9	2	2	0	0	0	3

**Table 3:** Scores of No. 3.

Consumers	Kansei indexes					
	Untechnical–technological	Inactive–dynamic	Outdated–futuristic	Feminine–manly	Dimmed–vivid	Partial–integral
1	2	3	2	2	2	3
2	2	2	2	3	3	3
3	3	2	1	−2	3	2
4	2	2	3	2	2	3
5	2	2	2	−1	3	2
6	3	3	1	0	2	2
7	2	2	2	3	2	1
8	2	2	2	2	1	2
9	1	3	−1	−1	2	3

**Table 4:** Consensus and deviation of viable solutions against No. 1 using PSO.

	Solution 1	Solution 2	Solution 3	Solution 4	Solution 5
CON	0.728	0.778	0.704	0.731	0.717
de	0.135	0.131	0.064	0.221	0.181

**Table 5:** Optimal adjusted evaluation value of No. 1.

Consumers	Kansei indexes					
	Untechnical–technological	Inactive–dynamic	Outdated–futuristic	Feminine–manly	Dimmed–vivid	Partial–integral
1	2.00	2.92	1.79	2.68	2.05	−1.82
2	2.89	1.37	2.79	1.99	2.11	1.58
3	2.03	2.87	−0.36	−1.48	2.28	0.37
4	0.70	1.31	1.36	1.58	1.78	1.90
5	−2.98	2.67	2.38	2.44	2.94	−1.56
6	2.02	1.82	2.73	2.15	2.89	0.16
7	2.11	1.13	−1.27	2.63	2.43	2.10
8	2.00	1.37	2.56	−0.69	0.57	0.42
9	2.22	2.15	1.61	2.99	1.55	1.24

**Table 6:** Consensus and deviation of viable solutions against No. 2 using PSO.

	Solution 1	Solution 2	Solution 3	Solution 4	Solution 5	Solution 6
CON	0.852	0.753	0.778	0.728	0.749	0.769
de	0.122	0.225	0.168	0.149	0.223	0.235

**Table 7:** Optimal adjusted evaluation value of No. 2.

Consumers	Kansei indexes					
	Untechnical–technological	Inactive–dynamic	Outdated–futuristic	Feminine–manly	Dimmed–vivid	Partial–integral
1	−0.32	2.97	1.77	1.96	−1.44	2.33
2	1.30	1.94	0.64	2.42	−0.59	1.69
3	−0.89	2.17	0.26	1.44	−0.74	−0.20
4	0.80	0.32	1.64	0.87	−0.12	1.49
5	1.10	1.59	1.21	1.77	−1.40	0.50
6	−2.73	2.06	0.79	1.64	−1.68	1.42
7	−0.34	1.75	−0.47	1.09	0.54	2.85
8	0.13	1.44	−1.02	2.36	0.27	−0.44
9	1.02	2.12	−0.16	0.74	0.42	2.34

**Table 8:** Consensus value and score of each design solution.

	No. 1		No. 2		No. 3	
	Score	Consensus	Score	Consensus	Score	Consensus
Considering consensus	0.754	0.704	0.612	0.852	0.799	0.704
Not considering consensus	0.732	0.531	0.558	0.630	0.799	0.704

## References

[1] Yang Y., Chen D., Gu R., Gu Y., Yu S. (2016). Consumers' Kansei needs clustering method for product emotional design based on numerical design structure matrix and genetic algorithms. *Computational Intelligence and Neuroscience*.

[2] Chang Y.-M., Chen C.-W. (2016). Kansei assessment of the constituent elements and the overall interrelations in car steering wheel design. *International Journal of Industrial Ergonomics*.

[3] Nagamachi M. (1995). Kansei Engineering: a new ergonomic consumer-oriented technology for product development. *International Journal of Industrial Ergonomics*.

[4] Nagamachi M. (2002). Kansei engineering as a powerful consumer-oriented technology for product development. *Applied Ergonomics*.

[5] Nagamachi M., Karwowski W. Kansei engineering and kansei evaluation.

[6] Lévy P. (2013). Beyond kansei engineering: the emancipation of kansei design. *International Journal of Design*.

[7] Grimsæth K. (2005). *Kansei Engineering Linking Emotions and Product Features*.

[8] Pambudi A. T., Suryoputro M. R., Sari A. D., Kurnia R. D. Design of Lesehan chair by using Kansei engineering method and anthropometry approach.

[9] Shergian A., Immawan T. (2015). Design of innovative alarm clock made from bamboo with kansei engineering approach. *Agriculture and Agricultural Science Procedia*.

[10] Razza B., Paschoarelli L. C. (2015). Affective perception of disposable razors: a kansei engineering approach. *Procedia Manufacturing*.

[11] Chuan N. K., Sivaji A., Shahimin M. M., Saad N. (2013). Kansei engineering for e-commerce sunglasses selection in Malaysia. *Procedia—Social and Behavioral Sciences*.

[12] Djatna T. K., Kurniati W. D. (2015). A system analysis and design for packaging design of powder shaped fresheners based on Kansei engineering. *Procedia Manufacturing*.

[13] Chen M.-C., Hsu C.-L., Chang K.-C., Chou M.-C. (2015). Applying Kansei engineering to design logistics services—a case of home delivery service. *International Journal of Industrial Ergonomics*.

[14] Huang M.-S., Tsai H.-C., Huang T.-H. (2011). Applying Kansei engineering to industrial machinery trade show booth design. *International Journal of Industrial Ergonomics*.

[15] Tharangie K. G. D., Irfan C. M. A., Yamad K., Marasinghe A. (2010). Kansei colour concepts to improve effective colour selection in designing human computer interfaces. *International Journal of Computer Science Issues (IJCSI)*.

[16] Wellings T., Williams M., Tennant C. (2010). Understanding customers' holistic perception of switches in automotive human-machine interfaces. *Applied Ergonomics*.

[17] Nevins J. L., Whitney D. E., De Fazio T. L. (1989). *Concurrent Design of Products and Processes: A Strategy for the Next Generation in Manufacturing*.

[18] Pahl G., Beitz W., Feldhusen J., Grote K.-H. (2007). *Engineering Design: A Systematic Approach*.

[19] Cross N. (2000). *Engineering Design Methods: Strategies for Product Design*.

[20] Chou J.-R. (2016). A Kansei evaluation approach based on the technique of computing with words. *Advanced Engineering Informatics*.

[21] Liao H., Xu Z., Zeng X.-J., Xu D.-L. (2016). An enhanced consensus reaching process in group decision making with intuitionistic fuzzy preference relations. *Information Sciences*.

[22] Mata F. M., Carlos J., Rodríguez R. (2011). *A Web-Based Consensus Support System Dealing with Heterogeneous Information*.

[23] González-Arteaga T., Alcantud J. C. R., de Andrés Calle R. (2016). A new consensus ranking approach for correlated ordinal information based on Mahalanobis distance. *Information Sciences*.

[24] Kacprzyk J., Fedrizzi M. (1988). A 'soft' measure of consensus in the setting of partial (fuzzy) preferences. *European Journal of Operational Research*.

[25] Zhang B., Dong Y., Xu Y. (2014). Multiple attribute consensus rules with minimum adjustments to support consensus reaching. *Knowledge-Based Systems*.

[26] Kennedy J. E., Russell C. (2001). *Swarm Intelligence*.

[27] Shi Y H., Eberhart R. C. Parameter selection in particle swarm optimization.

[28] Saaty T. L. (1980). *The Analytic Hierarchy Process: Planning, Priority Setting*.

[29] Eberhart R., Kennedy J. A new optimizer using particle swarm theory.

